# Reduced serum albumin as a risk factor for poor prognosis in critically ill patients receiving renal replacement therapy

**DOI:** 10.1186/s12882-021-02512-w

**Published:** 2021-09-08

**Authors:** Lang Jing Zheng, Weiming Jiang, Lingling Pan, Jingye Pan

**Affiliations:** Department of Intensive Care Unit, The First Affiliated Hospital of Wenzhou Medical University, Wenzhou, 325000 Zhejiang Province People’s Republic of China

**Keywords:** Albumin, Acute kidney injury, Continuous renal replacement therapy, Mortality, Subgroup

## Abstract

**Background:**

Albumin is the primary body protein, which can predict the poor prognosis of several critical diseases. However, there are a few scientific studies on the relationship between albumin and the prognosis of dialysis patients. This study aims to explore the impact of hypoalbuminemia on the prognosis of critically ill patients with acute kidney injury (AKI) receiving continuous renal replacement therapy (CRRT).

**Methods:**

This was a secondary study. Clinical, biochemical, and 28-day and 90-day mortality rates for critical patients with AKI who received CRRT between 2009 and 2016 were searched from the database to determine the effect of hypoalbuminemia on poor outcomes by univariate, multivariate, smooth curve fitting, and subgroup analysis.

**Results:**

A total of 837 participants were enrolled in this study. Multivariate Cox proportional hazard regression analysis showed that hypoalbuminemia was associated with both 28-day and 90-day mortality risks after full adjustment for confounding variables, with an adjusted hazard ratio (95% confidence interval) of 0.63 (0.50–0.80) and 0.63 (0.51–0.78), respectively for each 1 g/dL increase of albumin. Stratified analysis showed that hypoalbuminemia was not associated with poor prognosis in oliguria.

**Conclusion:**

Hypoalbuminemia is associated with poor prognosis in critically ill AKI patients with CRRT; therefore, measuring albumin may be helpful for predicting the prognosis. However, in those with oliguria, this conclusion is not valid.

## Introduction

Acute kidney injury (AKI) is a health challenge worldwide [1]. More than 13 million individuals get affected by AKI annually, which occurs in more than 60% of intensive care unit (ICU) patients [2]. Approximately 5% of ICU patients require continuous renal replacement therapy (CRRT) [3]. When the internal environment of patients with AKI is unstable, CRRT can easily control the imbalance. Therefore, CRRT is the most commonly used clinical renal replacement therapy [4, 5]. The mortality of patients with AKI is quite high, particularly in those with severe AKI who require dialysis; the mortality can exceed 50% [3]. Some clinical indicators, such as platelet, bilirubin, phosphate, Sequential Organ Failure Assessment (SOFA) score, and Acute Physiology and Chronic Health Evaluation II (APACHE II) score, have reportedly been related to the prognosis of critically ill patients with AKI [6–9].

Albumin is an important protein in the human body, which mainly exists in the blood, cerebrospinal, and lymphatic fluids. Albumin can not only maintain body nutrition and osmotic pressure but also combine with many insoluble substances, regulate the fluid exchange between various body parts, and affect blood circulation to the kidneys [10, 11]. Albumin reduction can increase the rate of mortality in acute coronary syndrome, septic shock, heart failure, and other diseases [12–14]. The albumin decrease is caused by the body in the inflammatory state. When the body is damaged, inflammation will occur, resulting in the high expression of platelet-derived growth factor, interleukin-6, and nitric oxide-induced vascular endothelial growth factor, which increase capillary permeability and angiogenesis, leading to the escape of albumin from the blood vessels [15, 16]. Gradually decreasing serum albumin levels possibly lead to more severe inflammation [17]. Inflammation plays an important role in the development of AKI [18]. Current studies have shown that serum albumin level is associated with an increased incidence rate of AKI and mortality in such patients [19, 20]. However, since the relationship between albumin levels and dialysis patient outcomes is controversial with only a few relevant studies [21, 22]; therefore, this study aimed to investigate the relationship between hypoalbuminemia and prognosis in patients with critical AKI under CRRT. The present study further investigated whether albumin affects mortality in specific populations with different CRRT indications.

## Materials and methods

### Data source

This study was a secondary analysis, with data from the ‘DATADRYAD’open database. The data packages uploaded by the researchers in the database can be downloaded and quoted for free. This study refers to the data package uploaded by Jung et al. [23, 24].

### Study population

Initially, a total of 2391 patients with AKI were enrolled in the study, all of whom received CRRT from January 2009 to September 2016 in the ICU of Yonsei University Health System Severance Hospital and the National Health Insurance Service Medical Center Ilsan Hospital. All participants met the Acute Kidney Injury Network criteria [25]. After analyzing the data of all participants, 281 with stage 1 AKI and 298 with stage 2 AKI were excluded. Also, the following populations were excluded: (1) minor patients (*n* = 42); (2) those with chronic kidney disease (CKD) or dialysis or CRRT history (*n* = 585); (3) those who were pregnant or lactating (*n* = 12); (4) those with retrorenal obstruction (*n* = 263); (5) those with renal transplantation history (*n* = 64); and (6) those with albumin deficiency (*n* = 9). Finally, 837 patients were included in this study.

In the study published in the original data [23], Jung et al. showed that the study followed the declaration of Helsinki and was approved by the Yonsei University health system, Severance Hospital, and Institutional Review Committee. Owing to the de-identified data, the patient’s written consent requirement was abandoned.

### Data collection

This study collected demographic and clinical data of patients before CRRT, including age, sex, body mass index (BMI), comorbidities, and CRRT causes. The main causes of AKI, including sepsis, nephrotoxin, ischemia, surgery and so on, were also listed in this paper. The laboratory indices recorded at 0 h of CRRT in the ICU were C-reactive protein (CRP), glomerular filtration rate (GFR), hemoglobin (Hb), white blood cell (WBC), serum creatinine (CR), blood urea nitrogen (BUN), albumin (Alb), bicarbonate (HCO3-), potassium (K +), and phosphate. Also, scores on the severity of the patient’s disease were collected, such as the Charlson comorbidity index (CCI), SOFA score, and APACHE II score.

### CRRT protocol

Clinically, doctors would decide whether to initiate CRRT according to the degree of renal function damage in critically ill patients. Patients with uremia, persistent oliguria, volume overload, and decompensated metabolic acidosis should start CRRT as soon as possible. The femoral, internal jugular, and subclavian veins were selected as the central venous catheterization sites of CRRT. The multiFiltrate (Fresenius Medical Care, Bad Homburg, Germany) or the Prismaflex (Baxter International Inc. Lundia AB, Sweden) machine was used for all patients. The initial CRRT blood flow velocity was 100 mL/min, which was increased to 150 mL/min. The total outflow of CRRT in all patients was targeted to deliver≥35 mL/kg/h.

### Statistical analysis

Continuous variables following normal distribution were expressed as the mean ± standard deviation, and skew variables were expressed as the median (quartile 1–quartile 3). Categorical variables were reported as the frequency or percentage. Box chart was used to show the relationship between serum albumin and different survival status, and analysis of variance was used to evaluate the difference between the survival and death groups. A univariate Cox proportional hazards model was established to analyze the risk relationship between albumin and 28-day and 90-day mortality in critically ill patients. Then, the multivariate Cox regression model was used to determine the independent effect of albumin on short-term and medium-term prognosis in critically ill patients. To ensure the robustness of the analysis results, this study not only gives the results of unadjusted, slight adjustment, and comprehensive adjustment analyses, but also converts albumin into classified variables for analysis according to the quartile method, and calculates the *p*-value of the trend to observe the possibility of nonlinearity. According to the indication of CRRT and the etiology of AKI, subgroup analysis was performed to further determine the relationship between albumin and short-term and medium-term prognosis. In order to explore whether there is a nonlinear relationship between albumin and prognosis, after adjusting all covariates, the correlation fitting curve between albumin and 28 and 90 day mortality was drawn by using the restrictive cubic spline function following on Cox proportional hazards models. In this study, *P* < 0.05 was considered a significant difference.

In the present study, the variable CCI was transformed into continuous variable processing, and all data were processed using EmpowerStats (www.empowerstats.com, X&Y solutions, inc. Boston, Massachusetts) and statistical packages R (http://www.R-project.org; The R Foundation; version 3.4.3).

## Results

### Baseline characteristics of participants

**Fig. 1:**
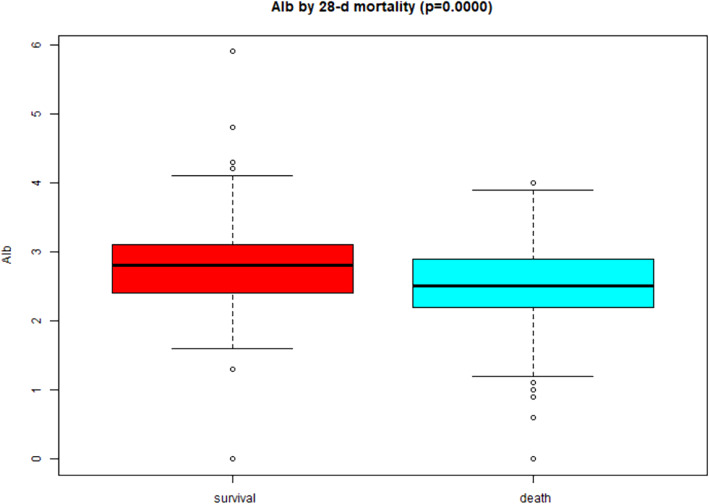
Boxplot exploring the relationship between serum albumin and different survival status (28-d mortality)

**Fig. 2:**
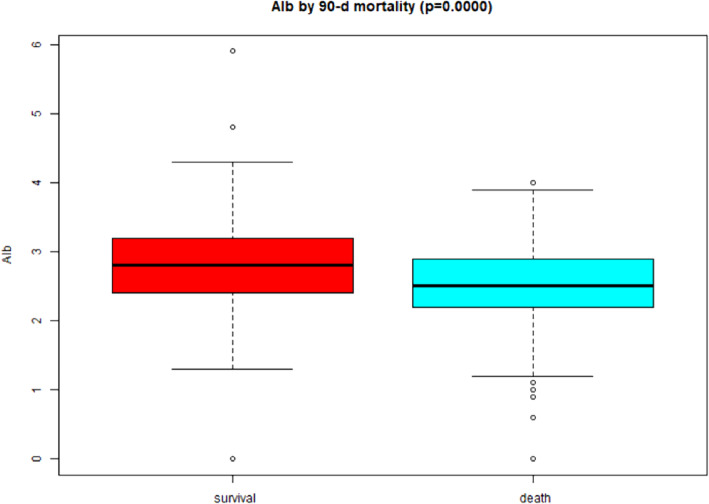
Boxplot exploring the relationship between serum albumin and different survival status (90-d mortality)

Table 1 lists the general characteristics of the included participants. The average age of the study participants was 62.57 ± 14.42 years, and 62.25% were men. The mean BMI and mean arterial pressure (MAP) were 23.90 ± 4.83 kg/m2 and 76.78 ± 14.51 mmHg, respectively. Most of the critically ill patients had complications, including 406 (48.51%) patients with hypertension, 266 (31.82%) with diabetes, 112 (13.38%) with congestive heart failure, and 90 (10.78%) patients with cerebrovascular disease. Also, the prevalence of peripheral vascular disease, chronic obstructive pulmonary disease (COPD), myocardial infarction, and dementia was 29 (3.46%), 51 (6.09%), 75 (8.96%), and 26 (3.11%), respectively. Among the 837 participants, the 28-day and the 90-day mortality rate was 61.89 and 71.92%, respectively. The box chart showed that serum albumin level was correlated with different survival statuses of 28 days and 90 days, and the serum albumin level of the survival group was significantly higher than that of the death group (both *p* < 0.001) (Figs. 1 and 2).

**Table 1:** Baseline characteristics of participants

Characteristics	Values
Age, year	62.57 ± 14.42
SEX (male/female)	521/316
BMI, kg/m2	23.90 ± 4.83
MAP, mmHG	76.78 ± 14.51
Myocardial infarction, n (%)	75 (8.96%)
Congestive heart failure, n (%)	112 (13.38%)
Cerebrovascular disease, n (%)	90 (10.78%)
Peripheral vascular disease, n (%)	29 (3.46%)
Dementia, n (%)	26 (3.11%)
Diabetes, n (%)	266 (31.82%)
Hypertension, n (%)	406 (48.51%)
COPD, n (%)	51 (6.09%)
Mechanical ventilation, n (%)	661 (78.97%)
K+, mEq/L	4.72 ± 1.10
HCO3-, mEq/L	16.54 ± 5.65
Phosphate, g/dL	5.94 ± 2.53
CRP, g/L	66.45 (18.88–164.80)
WBC, μL	11,840.00 (6565.00–18,672.50)
HBIG/dL	9.66 ± 2.28
Albago/dL	2.61 ± 0.59
BUN, g/dL	52.00 (35.00–76.00)
Cr, mg/dL	3.02 ± 1.74
GFR, mL/min	23.30 (15.10–35.80)
APACHE II score	27.58 ± 7.89
SOFA score	12.53 ± 3.50
CCI	3.13 ± 2.25
AKI causes	
Sepsis	580 (69.30%)
Nephrotoxin	26 (3.11%)
Ischemia	65 (7.77%)
Surgery	70 (8.36%)
Others	96 (11.47%)
CRRT causes	
Volume overload	108 (12.90%)
Metabolic acidosis	183 (21.86%)
Hyperkalemia	39 (4.66%)
Uremia	93 (11.11%)
Oliguria	219 (26.16%)
Other	195 (23.30%)
28-d mortality	518 (61.89%)
90-d mortality	602 (71.92%)

Data are expressed as n (%) or the mean ± SD or median (quartile 1-quartile 3)

*SD* Standard deviation

### Univariate analysis

As shown in Tables 2 and 3, Univariate analysis showed that albumin was associated with 28-day and 90-day mortality in critically ill patients. The hazard ratio (HR) and 95% confidence interval (CI) were 0.66 (0.57, 0.76) and 0.62 (0.55, 0.71), respectively. Albumin was a protective factor. Also, the univariate analysis found that hypertension, APACHE II score, SOFA score, CCI, phosphate, MAP, mechanical ventilation, Hb, Cr, and GFR were associated with short-term and medium-term prognosis of critically ill AKI patients with CRRT, while BMI was only associated with 90-day mortality.

**Table 2:** The results of univariate analysis of 28-day mortality

Variable	HR (95%CI)	*p*-value
Age	1.00 (0.99, 1.01)	0.9847
SEX		
male	1.0	
female	0.94 (0.78, 1.12)	0.4792
Myocardial infarction	0.99 (0.74, 1.33)	0.9567
Congestive heart failure	0.80 (0.61, 1.04)	0.0947
Cerebrovascular disease	0.90 (0.68, 1.19)	0.4633
Peripheral vascular disease	0.76 (0.46, 1.25)	0.2844
Dementia	0.71 (0.42, 1.20)	0.1998
Diabetes	0.91 (0.76, 1.10)	0.3334
Hypertension	0.69 (0.58, 0.83)	< 0.0001
COPD	0.91 (0.62, 1.32)	0.6039
K+	1.03 (0.95, 1.12)	0.4208
APACHE II score	1.03 (1.02, 1.04)	< 0.0001
CCI	1.10 (1.06, 1.14)	< 0.0001
SOFA score	1.17 (1.14, 1.21)	< 0.0001
CRRT causes		
Volume overload	1.0	
Metabolic acidosis	1.45 (1.06, 1.99)	0.0207
Hyperkalemia	1.66 (1.05, 2.61)	0.0285
Uremia	0.94 (0.65, 1.38)	0.7674
Oliguria	1.08 (0.78, 1.47)	0.6515
Other	1.45 (1.06, 1.98)	0.0199
AKI causes		
Sepsis	1.0	
Nephrotoxin	1.06 (0.66, 1.70)	0.8108
Ischemia	1.06 (0.76, 1.47)	0.7251
Surgery	0.81 (0.58, 1.15)	0.2370
Others	1.32 (1.00, 1.72)	0.0464
HCO3-	0.99 (0.97, 1.01)	0.3030
Phosphate	1.05 (1.02, 1.08)	0.0018
BMI	0.98 (0.96, 1.00)	0.0626
MAP	0.98 (0.98, 0.99)	< 0.0001
Mechanical ventilation	2.12 (1.65, 2.73)	< 0.0001
WBC	1.00 (1.00, 1.00)	0.1276
Hb	0.93 (0.90, 0.97)	0.0004
BUN	1.00 (1.00, 1.00)	0.8286
Cr	0.89 (0.84, 0.94)	< 0.0001
Alb	0.66 (0.57, 0.76)	< 0.0001
CRP	1.00 (1.00, 1.00)	0.8429
GFR	1.00 (1.00, 1.01)	0.0256

**Table 3:** The results of univariate analysis of 90-day mortality

Variable	HR (95%CI)	*p*-value
Age	1.00 (1.00, 1.01)	0.4307
SEX		
male	1.0	
female	0.96 (0.82, 1.14)	0.6630
Myocardial infarction	0.93 (0.70, 1.24)	0.6179
Congestive heart failure	0.90 (0.71, 1.14)	0.3679
Cerebrovascular disease	1.02 (0.79, 1.33)	0.8560
Peripheral vascular disease	0.94 (0.60, 1.47)	0.7887
Dementia	0.87 (0.53, 1.40)	0.5580
Diabetes	0.87 (0.73, 1.04)	0.1202
Hypertension	0.72 (0.61, 0.84)	< 0.0001
COPD	0.94 (0.67, 1.32)	0.7179
K+	1.07 (0.99, 1.15)	0.0922
APACHE II score	1.03 (1.02, 1.04)	< 0.0001
CCI	1.07 (1.04, 1.11)	< 0.0001
SOFA score	1.13 (1.10, 1.15)	< 0.0001
CRRT causes		
Volume overload	1.0	
Metabolic acidosis	1.41 (1.05, 1.87)	0.0205
Hyperkalemia	1.78 (1.18, 2.70)	0.0063
Uremia	1.08 (0.77, 1.52)	0.6628
Oliguria	1.14 (0.86, 1.52)	0.3584
Other	1.32 (0.99, 1.75)	0.0596
AKI causes		
Sepsis	1.0	
Nephrotoxin	0.96 (0.60, 1.54)	0.8664
Ischemia	1.01 (0.75, 1.37)	0.9277
Surgery	0.67 (0.49, 0.93)	0.0151
Others	0.90 (0.70, 1.17)	0.4399
HCO3-	0.99 (0.98, 1.01)	0.4557
Phosphate	1.04 (1.01, 1.08)	0.0054
BMI	0.98 (0.96, 1.00)	0.0163
MAP	0.98 (0.98, 0.99)	< 0.0001
Mechanical ventilation	1.97 (1.58, 2.46)	< 0.0001
WBC	1.00 (1.00, 1.00)	0.2207
Hb	0.93 (0.90, 0.97)	0.0002
BUN	1.00 (1.00, 1.00)	0.5005
Cr	0.89 (0.84, 0.93)	< 0.0001
Alb	0.62 (0.55, 0.71)	< 0.0001
CRP	1.00 (1.00, 1.00)	0.8145
GFR	1.01 (1.00, 1.01)	0.0003

### Multivariate cox regression analysis

To further clarify the relationship between albumin and short-term and medium-term prognosis, we established three models of unadjusted, micro adjusted, and fully adjusted in multivariate regression analysis. The results of the unadjusted model (model 1) showed that albumin was negatively correlated with 28-day and 90-day mortality. In model 2 (adjusted for age and sex only), the risks of 28-day and 90-day mortality decreased by 34 and 38%, respectively for every 1 g/dL increase in albumin. After adjusting all the covariates (model 3), the HR (95% CI) of 28-day and 90-day mortality was 0.63 (95% CI 0.50–0.80, *p* = 0.0001) and 0.63 (95% CI 0.51–0.78, *p* < 0.0001), respectively for each 1 g/dL increase of albumin. To explore the nonlinearity of albumin regarding 28-day and 90-day mortality of critically ill AKI patients with CRRT, we also converted albumin into categorical variables (quartile) and found that P for the trend of albumin with 28-day and 90-day mortality of the patients was less than 0.01 (Table 4).

**Table 4:** The results of multivariate Cox proportional hazards regression analysis

Variable	28-d mortality (HR 95%CI, p)	90-d mortality (HR 95%CI, p)
Model 1		
Alb	0.66 (0.57, 0.76) < 0.0001	0.62 (0.55, 0.71) < 0.0001
Alb(quartile)		
Q1	1.0	1.0
Q2	0.93 (0.73, 1.18) 0.5395	0.84 (0.67, 1.05) 0.1222
Q3	0.75 (0.59, 0.96) 0.0219	0.69 (0.55, 0.86) 0.0012
Q4	0.56 (0.43, 0.72) < 0.0001	0.49 (0.38, 0.62) < 0.0001
P for trend	< 0.0001	< 0.0001
Model 2		
Alb	0.66 (0.57, 0.76) < 0.0001	0.62 (0.55, 0.71) < 0.0001
Alb(quartile)		
Q1	1.0	1.0
Q2	0.92 (0.73, 1.17) 0.5021	0.84 (0.68, 1.05) 0.1289
Q3	0.75 (0.59, 0.96) 0.0220	0.69 (0.55, 0.86) 0.0012
Q4	0.55 (0.43, 0.72) < 0.0001	0.49 (0.38, 0.62) < 0.0001
P for trend	< 0.0001	< 0.0001
Model 3		
Alb	0.63 (0.50, 0.80) 0.0001	0.63 (0.51, 0.78) < 0.0001
Alb(quartile)		
Q1	1.0	1.0
Q2	0.71 (0.50, 1.02) 0.0622	0.66 (0.47, 0.92) 0.0152
Q3	0.62 (0.44, 0.88) 0.0076	0.63 (0.46, 0.87) 0.0054
Q4	0.52 (0.35, 0.76) 0.0008	0.52 (0.37, 0.74) 0.0003
P for trend	0.0007	0.0006

Model 1 adjust for: none

Model 2 adjust for: age; sex

Model 3 adjust for: age, sex, myocardial infarction, congestive heart failure, cerebrovascular disease, peripheral vascular disease, dementia, diabetes, hypertension, COPD, CRP, GFR, K+, HCO3-, phosphate, BMI, MAP, WBC, Hb, BUN, Cr, mechanical ventilation, CCI, APACHE II score, SOFA score, CRRT causes, AKI causes

Q1:0.00–2.10, Q2:2.20–2.50, Q3:2.60–2.90, Q4:3.00–5.90

### The results of subgroup analyses

A subgroup analysis was performed according to the CRRT indications. In the subgroup analysis based on the reasons for using CRRT, albumin was found to have a protective effect on the prognosis of patients with volume overload and metabolic acidosis. In patients with volume overload, the HR (95% CI) of 28-day and 90-day mortality were 0.10 (95% CI 0.02–0.39, *p* < 0.001) and 0.15 (95% CI 0.05–0.43, p < 0.001), respectively for each 1 g/dL increase in albumin after adjusting for all covariates. In patients with metabolic acidosis, the risks of 28-day and 90-day mortality decreased with the increasing albumin, and the adjusted HR (95% CI) was 0.24 (95% CI 0.11–0.51, p < 0.001) and 0.28 (95% CI 0.15–0.53, p < 0.001), respectively (Table 5).

**Table 5:** The results of subgroup analysis based on continuous renal replacement therapy (CRRT) causes

Variable	28-d mortality (HR 95%CI, p)	90-d mortality (HR 95%CI, p)
Volume overload		
Model 1		
Alb	0.48 (0.31, 0.76) 0.0018	0.45 (0.28, 0.71) 0.0006
Model 2		
Alb	0.47 (0.29, 0.75) 0.0016	0.45 (0.28, 0.71) 0.0006
Model 3		
Alb	0.10 (0.02, 0.39) 0.0009	0.15 (0.05, 0.43) 0.0005
Metabolic acidosis		
Model 1		
Alb	0.59 (0.44, 0.80) 0.0006	0.61 (0.47, 0.80) 0.0003
Model 2		
Alb	0.62 (0.46, 0.84) 0.0017	0.63 (0.49, 0.83) 0.0008
Model 3		
Alb	0.24 (0.11, 0.51) 0.0002	0.28 (0.15, 0.53) 0.0001
Oliguria		
Model 1		
Alb	0.78 (0.60, 1.03) 0.0785	0.76 (0.59, 0.96) 0.0227
Model 2		
Alb	0.79 (0.61, 1.04) 0.0974	0.76 (0.60, 0.97) 0.0290
Model 3		
Alb	1.02 (0.60, 1.73) 0.9304	0.89 (0.56, 1.41) 0.6301
Hyperkalemia		
Model 1		
Alb	0.66 (0.37, 1.20) 0.1734	0.66 (0.39, 1.12) 0.1208
Model 2		
Alb	0.70 (0.39, 1.28) 0.2514	0.71 (0.41, 1.22) 0.2104
Model 3		
Alb	---^a^	---^a^
Uremia		
Model 1		
Alb	0.54 (0.30, 0.95) 0.0316	0.33 (0.19, 0.58) 0.0001
Model 2		
Alb	0.49 (0.27, 0.91) 0.0244	0.33 (0.19, 0.59) 0.0001
Model 3		
Alb	---^a^	---^a^
Other		
Model 1		
Alb	0.73 (0.54, 0.98) 0.0386	0.66 (0.50, 0.87) 0.0032
Model 2		
Alb	0.74 (0.55, 1.00) 0.0467	0.65 (0.49, 0.87) 0.0037
Model 3		
Alb	0.44 (0.25, 0.79) 0.0058	0.43 (0.25, 0.74) 0.0022

Model 1 adjust for: none

Model 2 adjust for: age; sex

Model 3 adjust for: age, sex, myocardial infarction, congestive heart failure, cerebrovascular disease, peripheral vascular disease, dementia, diabetes, hypertension, COPD, CRP, GFR, K+, HCO3-, phosphate, BMI, MAP, WBC, Hb, BUN, Cr, mechanical ventilation, CCI, APACHE II score, SOFA score, AKI causes

^a^: The model failed because of the small sample size

### Curve fitting analysis

**Fig. 3:**
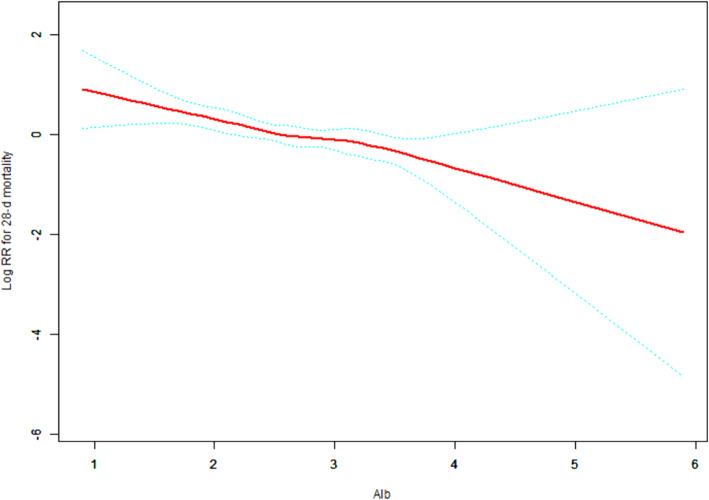
Adjusted smoothing function of albumin for 28-d mortality

**Fig. 4:**
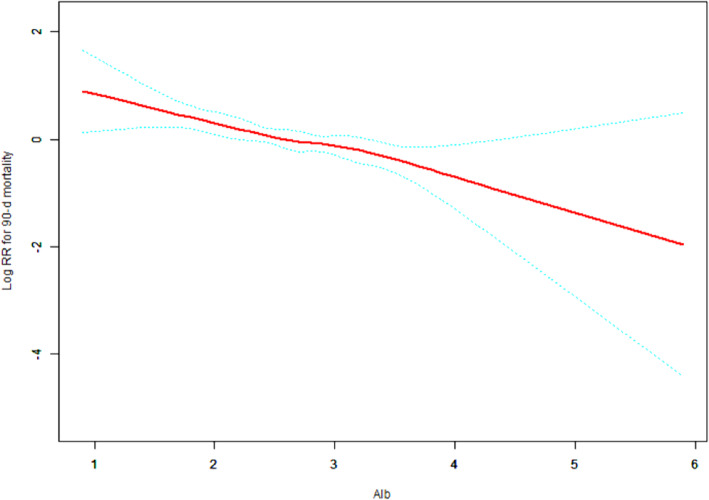
Adjusted smoothing function of albumin for 90-d mortality

To investigate the relationship between albumin and prognosis in CRRT patients with critical AKI, we performed a fitting curve analysis. After adjusting age, sex, myocardial infarction, congestive heart failure, cerebrovascular disease, peripheral vascular disease, dementia, diabetes, hypertension, COPD, CRP, GFR, K+, HCO3-, phosphate, BMI, MAP, WBC, Hb, BUN, Cr, mechanical ventilation, CCI, APACHE II score, SOFA score, CRRT causes, and AKI causes, it was found that with the increasing albumin, the risks of 28-day and 90-day mortality of patients decreased (*p* < 0.001) (Figs. 3 and 4).

## Discussion

In this study, a negative correlation was depicted between albumin and the risks of 28-day and 90-day mortality in critically ill AKI patients with CRRT using a fully adjusted model. Also, the relationship between albumin and prognosis was different in patients with CRRT due to different causes.

Protein is the basic component of supporting life, and albumin accounts for approximately 40–60% of the total protein content in the body. It is primarily synthesized by the hepatocytes. It can regulate plasma colloidal osmotic pressure, maintain the blood vessel barrier, and act as a carrier to transport insoluble substances [26–28]. The decrease of plasma albumin is caused by several pathological mechanisms, including decreased synthesis, increased decomposition, and redistribution of albumin [29]. Acute and chronic inflammation can affect liver protein metabolism through the inflammatory mechanism, reducing albumin synthesis and inducing an increase in capillary permeability; thus, reducing plasma albumin levels [16, 30]. It is believed that the increase of albumin catabolism in hemodialysis patients induced by inflammation leads to hypoalbuminemia [17].

Thus, inflammation plays an important role in the occurrence and development of AKI [31]. AKI can lead to long-term effects of renal fibrosis and chronic inflammation and further progress to CKD [32, 33]. Also, albumin has a large number of reduced sulfhydryl groups; therefore, it has an antioxidant effect, which in excess can lead to kidney damage. Therefore, patients with reduced albumin are prone to AKI and CKD; thus, kidney function cannot recover in a short period and will increase the risk of death [30, 34].

Several studies have shown that the decrease in serum albumin can predict the poor prognosis of the disease. A study by Olivia et al. in 486 severely burned patients demonstrated that albumin was a good predictor of burn mortality, with a reported area under the curve of 0.869, and that the death risk increased by more than 80% when albumin level was below 2 g/dL [35]. In Reza Behrouz’s study, hypoalbuminemia was found to be independently associated with in-hospital mortality in patients with aneurysms subarachnoid hemorrhage, with an adjusted odds ratio (95% CI) of 4.26 (95% CI: 1.09–16.68, *p* = 0.04) [36]. This study shows that albumin levels are associated with prognosis in critical patients with AKI under CRRT, which is consistent with the results of previous studies by Moon et al. [22]. However, more confounding variables were adjusted in this study to make the results more reliable. Simultaneously, the sensitivity analysis was also performed in the multivariate Cox regression analysis, and the nonlinear relationship between albumin and prognosis was observed to ensure the robustness of the results. Subgroup analysis based on CRRT indications demonstrated that some results were not significant or missing; however, the general trend was consistent. The subgroup possibly included fewer samples, which reduced the ability of the statistical system. Additionally, we observed that albumin was not associated with the risk of death in patients with oliguria. It is well-established that when renal function is impaired, the number of perirenal capillaries is reduced, and renal blood flow is greatly decreased, leading to reduced urine volume in patients [37]. Oliguria is a risk factor for poor prognosis in dialysis patients [21]. In patients with severe oliguria or anuria, the effect of decreased albumin on prognosis may be ignored. However, more research is needed to confirm the results.

There were some limitations in this study: 1) Our data were from Dryad’s open database, and knowing whether patients received albumin infusion before CRRT was infeasible, which could affect albumin levels; thus, skewing the results; 2) Due to the limitations of the database, we could not know whether patients classified according to the etiology of AKI were mutually exclusive. So, the findings of this study needed to be further confirmed by large prospective studies.

## Conclusions

In this study, we investigated the predictive value of hypoproteinemia in critical CRRT patients with poor prognoses. We also observed that albumin was not associated with prognosis in patients with oliguria.

## Data Availability

The data are obtained from the ‘DataDryad’ database (www.datadryad.org).

## Abbreviations

AKI: Acute kidney injury
CRRT: Receiving renal replacement therapy
CI: Confidence interval
HR: Hazard ratio
ICU: Intensive care unit
IL: Interleukin
NO: Nitric oxide
VEGF: Vascular endothelial growth factor
AKIN: Acute Kidney Injury Network
BMI: Body mass index
CRP: C-Reactive protein
GFR: Glomerular filtration rate
HB: Hemoglobin
WBC: White blood cell
CR: Serum creatinine
BUN: Blood urea nitrogen
Alb: Albumin
HCO3-: Bicarbonate
K +: Potassium
CCI: Charlson comorbidity index
SOFA: Sequential Organ Failure Assessment
APACHE II: Acute Physiology and Chronic Health Evaluation II
SD: Standard deviation
COPD: Chronic obstructive pulmonary disease
